# Benign Acute Childhood Myositis Before and After COVID-19: A Nine-Year Retrospective Study

**DOI:** 10.3390/pediatric18020047

**Published:** 2026-03-31

**Authors:** Helena Ferreira, Carolina Pinto da Costa, Sofia Silva Faria, Ana Luísa Correia, Sofia Aroso

**Affiliations:** 1 Pediatric Department, Hospital Pedro Hispano, ULS Matosinhos, 4464-513 Matosinhos, Portugal; 2 Pediatric Department, Centro Materno-Infantil do Norte, ULS Santo António, 4050-651 Porto, Portugal

**Keywords:** COVID-19, influenza, myositis

## Abstract

**Background/Objectives**: This aim of this study was to describe the demographic, clinical, and laboratory characteristics of hospitalized children with benign acute childhood myositis (BACM) and to evaluate seasonal patterns, including changes observed during the COVID-19 pandemic. **Methods**: We conducted a retrospective single-center review of pediatric patients hospitalized with a diagnosis of BACM between January 2016 and December 2024. Clinical, laboratory, and epidemiological data were analyzed, including seasonal distribution before and after the COVID-19 pandemic. **Results**: We identified 47 cases of BACM, with a male predominance (66%) and a median age of 7 years. Most cases (72%) occurred during autumn and spring. The most common prodromal symptoms were fever, cough and rhinorrhea. Bilateral calf pain was the most frequent presenting symptom. The median creatine phosphokinase (CPK) level was 4986 U/L, with higher values in boys (*p* = 0.040). Higher CPK levels were associated with longer hospital stays in our cohort (*p* = 0.030). Influenza B was the most frequently identified pathogen (63%). No BACM cases were recorded during the COVID-19 pandemic period (2020–2022), followed by an increase in 2024. All patients fully recovered, with a median hospital stay of 3.2 days. **Conclusions**: BACM is a self-limiting condition with a characteristic clinical and laboratory profile. The absence of cases during the COVID-19 pandemic suggests a possible association between reduced viral circulation and BACM incidence. Awareness of its typical presentation may support early diagnosis, reduce unnecessary investigations, and facilitate appropriate clinical management.

## 1. Introduction

Benign acute childhood myositis (BACM) is a transient, self-limited muscle condition predominantly associated with influenza A and B viruses; however, a broader spectrum of viral pathogens has also been implicated, including enteroviruses, adenovirus, parainfluenza viruses, respiratory syncytial virus, and herpes simplex virus [1,2]. First described by Lundberg in 1957 as *myalgia cruris epidemica*, the condition exhibits seasonal variation, with higher prevalence during colder months, particularly in winter and early spring [2,3]. The clinical presentation is characterized by the sudden onset of lower extremity pain and difficulty walking, usually following upper respiratory symptoms such as fever, cough, and rhinorrhea. Physical examination may reveal calf tenderness, while the neurological examination typically remains normal, with preserved ankle and knee reflexes, helping to distinguish BACM from other neuromuscular disorders [1,4]. Laboratory findings commonly include elevated serum creatine kinase (CPK) and aspartate aminotransferase (AST) levels, reflecting muscle inflammation and injury. Although the exact pathophysiological mechanisms remain unclear, both direct viral invasion and immune-mediated processes have been proposed [5,6].

Management of BACM is mainly supportive, including analgesia, adequate hydration, and rest. In selected cases, intravenous fluids and hospitalization may be required to promote the clearance of muscle breakdown products and prevent complications. Clinical and laboratory abnormalities typically resolve within one to two weeks, with most patients achieving full recovery without long-term sequelae [7,8,9,10]. Nevertheless, rare complications such as rhabdomyolysis may occur, with potential secondary acute kidney injury due to myoglobinuria [1,10].

Despite its favorable prognosis, BACM is often a source of significant concern for caregivers and healthcare providers, leading to extensive and frequently unnecessary diagnostic workups. Further evaluation for alternative diagnoses, including neuromuscular or metabolic disorders, should generally be reserved for atypical, recurrent, or severe presentations, particularly when CK levels are markedly elevated.

This study presents a nine-year retrospective analysis of all patients admitted to our pediatric department with a diagnosis of BACM. We aimed to describe the demographic, clinical, and laboratory characteristics of hospitalized cases, analyze seasonal patterns—including the impact of the COVID-19 pandemic—and explore associations between clinical features and outcomes.

## 2. Materials and Methods

We conducted a retrospective, single-center study of pediatric patients hospitalized with a diagnosis of BACM at a secondary hospital in Matosinhos, Portugal, between 1 January 2016 and 31 December 2024. Patients were identified through electronic medical records.

The diagnosis of BACM was established based on the presence of acute-onset bilateral calf pain and/or gait disturbance, elevated serum creatine kinase levels above age-adjusted reference ranges, a recent viral prodrome, and the absence of neurological deficits or alternative diagnoses after comprehensive clinical evaluation. Patients were admitted when at least one of the following criteria was present: severe pain and/or refusal to ambulate; serum creatine kinase levels greater than ten times the upper limit of normal (with values above 15,000 U/L indicating an increased risk of acute kidney injury); inadequate capacity for home monitoring by caregivers; or clinical and/or laboratory evidence of rhabdomyolysis. All patients younger than 18 years hospitalized with a diagnosis of BACM at our institution during the study period were included.

Demographic, clinical, and laboratory data, including age, sex, prodromal symptoms, presenting symptoms, CPK and myoglobin levels, inflammatory markers, length of hospital stay, therapeutic management, outcomes, and complications, were extracted from electronic medical records and analyzed. When available, results of viral testing performed using real-time polymerase chain reaction (PCR) or antigen detection assays on nasopharyngeal swabs were also recorded.

All statistical analyses were performed using SPSS^®^ software, version 22.0. Continuous variables were summarized as means with standard deviations (SDs) for normally distributed data and as medians with interquartile ranges (IQRs) for non-normally distributed data. Categorical variables were expressed as frequencies and percentages. Comparisons between independent groups for continuous non-parametric data were performed using the Mann–Whitney U test, while comparisons involving more than two independent groups were conducted using the Kruskal–Wallis test. Associations between continuous variables were assessed using Spearman’s rank correlation coefficient. To compare the number of admissions in 2024 with previous years, the Wilcoxon signed-rank test was used. A *p*-value < 0.05 was considered statistically significant.

## 3. Results

### 3.1. Epidemiology

A total of 47 hospital admissions for BACM were recorded between 1 January 2016, and 31 December 2024. Most patients were male (66%), with a mean (SD) age at admission of 7.3 (2.4) years. The mean (SD) length of hospital stay was 3.2 (1.1) days (Table 1).

Admissions exhibited seasonal variation, with 40.4% occurring in autumn (19 cases) and 31.9% in spring (15 cases), while winter and summer accounted for 21.3% and 6.4%, respectively. The highest number of cases occurred in May and December (23.4% each), whereas no cases were reported in July or August.

Regarding annual distribution, 2024 accounted for the highest number of cases (22 cases, 46.8%), exceeding the median number observed in previous years (Wilcoxon signed-rank test, *p* = 0.004). The second-highest peak occurred in 2019, with 14 cases. No BACM cases were recorded during the COVID-19 pandemic years (2020–2022). Despite the increase in cases in 2024, patient characteristics, including age, sex, length of stay, and CPK levels, remained similar to those observed in previous years.

### 3.2. Clinical Manifestations

The most common prodromal symptoms were fever (97.9%), cough (72.3%), rhinorrhea (63.8%), odynophagia (29.8%), diarrhea (10.6%), and vomiting (8.5%) (Table 1). Bilateral calf pain was the most common presenting symptom, affecting 95.7% of patients, followed by refusal to walk (59.6%) and gait abnormality (44.7%). No neurological abnormalities other than gait disturbances were observed. The median time from the onset of prodromal symptoms to the development of BACM symptoms was 3.0 days (IQR of 1.0). A negative correlation was found between this interval and CPK levels (Spearman’s rank correlation, *p* = 0.003), indicating that patients with a shorter prodromal period tended to have higher CPK levels.

### 3.3. Etiology

Viral testing was performed in 91.5% of patients (n = 43). Influenza B was the most frequently identified pathogen (63.8%), followed by influenza A (19.2%). Two patients with influenza B also tested positive for Mycoplasma pneumoniae. No specific viral agent was associated with higher CPK levels (Kruskal–Wallis test, *p* = 0.981) or longer hospital stay (Kruskal–Wallis test, *p* = 0.981).

### 3.4. Laboratory Findings

The median peak CPK value was 4986 U/L (IQR of 5489 U/L). Boys had higher CPK levels than girls (*p* = 0.040). A correlation was observed between CPK levels and duration of hospitalization (Spearman’s rank correlation, *p* = 0.030), indicating that patients with higher CPK values required longer inpatient care. Myoglobinemia was assessed in 79.6% of cases, with a median value of 680 ng/mL (IQR of 1407 ng/mL).

Elevated liver enzymes were common, with ALT elevated in 59.6% of patients (median of 51 U/L, IQR of 38 U/L) and AST elevated in 89.4% (median of 178 U/L, IQR of 167 U/L). LDH levels were evaluated in 48.9% of patients, with a median of 445 U/L (IQR of 167 U/L).

The most frequent hematological findings were leukopenia (40.4%) and thrombocytopenia (38.3%). C-reactive protein (CRP) levels were low, with a median of 1.45 mg/L (IQR of 4.25 mg/L). Only one patient had myoglobinuria, which resolved within 24 h with intravenous hydration. All patients had normal creatinine levels, and one patient had a mild elevation in urea, which also resolved within 24 h with intravenous hydration.

### 3.5. Evolution

All patients received intravenous hydration and analgesics as needed. Oseltamivir was administered to all patients with confirmed influenza infection. Complete recovery was observed in all cases, and none of the patients required admission to the intensive care unit. One patient experienced recurrence and was referred for metabolic disease evaluation, while another, with markedly elevated CPK levels (20,321 U/L), underwent further investigation to exclude underlying neuromuscular or metabolic disorders. The metabolic evaluation was normal. A neuromuscular gene panel identified two variants of uncertain significance, including one in the *FLNC* gene, previously associated with autosomal dominant myofibrillar and distal myopathies, findings not consistent with the patient’s clinical presentation. Genetic counseling for familial segregation analysis was declined by the family, and counseling was recommended for future reproductive planning.

## 4. Discussion

Benign acute childhood myositis (BACM) is a well-recognized transient muscle disorder associated with viral infections, primarily influenza A and B [2,9]. The underlying pathophysiological mechanisms remain incompletely understood, underscoring the need for further research to clarify the contributing factors and disease processes [9]. The findings of our study are consistent with the typical epidemiological and clinical characteristics of BACM, aligning with prior studies reporting a higher incidence in school-aged children, predominantly boys, and a seasonal pattern peaking in winter and early spring [2,10,11]. In our cohort, we observed a greater prevalence among males (31 boys vs. 16 girls), with higher median CPK levels in boys. The reasons for this male predominance remain unclear. Mackay et al. [5] hypothesized a possible genetic predisposition, whereas Rajajee et al. [12] suggested that higher physical activity levels in boys could be a contributing factor. BACM primarily affects children aged 5 to 9 years, with rare occurrences in adults [1,9]. Our study reflects this pattern, with a mean age of 7.25 years. This age-related prevalence may be explained by greater physical activity, reluctance to rest, and developmental aspects of skeletal muscle physiology [2]. There is no robust evidence demonstrating a different pattern of muscle group involvement in non-ambulatory children with BACM. Most available data derive from school-aged, ambulatory children, and reports in younger infants remain scarce and heterogeneous. Likewise, there is currently no robust evidence supporting a direct relationship between BACM and longitudinal growth.

The prodromal symptoms observed in our cohort—fever, cough, and rhinorrhea—were consistent with those reported in the literature, with a median interval of three days (IQR of 1) between the onset of prodromal symptoms and the development of muscle-related symptoms, similar to previous studies [4,11,13]. The primary clinical manifestations included lower limb pain, gait disturbances, and refusal to walk, all hallmark features of BACM [2]. Clinically, BACM most often presents with sudden-onset bilateral calf pain and gait difficulty, with preservation of strength and reflexes on examination. While unilateral calf pain has been described, it appears less common. In our cohort, calf pain was bilateral in 95.7%, reinforcing the typical bilateral pattern in hospitalized cases. Importantly, neurological examination was unremarkable beyond walking difficulties, emphasizing the need for careful medical history and clinical evaluation to differentiate BACM from other causes of gait disturbances, particularly in cases with unilateral complaints, where alternative neurological or orthopedic diagnoses should be considered [2,4].

Laboratory findings demonstrated elevated CPK levels, with a median of 4986 U/L (IQR of 5488.5 U/L), which is slightly higher than reported in other studies [10,14,15]. This difference may be partly explained by the inclusion of exclusively hospitalized patients in our cohort, whereas some previous studies also included non-hospitalized children with milder presentations. CK/CPK values may differ across laboratories due to analytical and pre-analytical factors, including assay principles (commonly IFCC-aligned kinetic methods), analyzer calibration and traceability, and temperature standardization. In addition, pre-analytical sample issues, particularly hemolysis, may interfere with enzyme activity measurements and contribute to inter-laboratory variability [16]. Therefore, absolute CK values should be interpreted in the clinical context and, when monitoring trends, ideally using the same laboratory method over time, as performed in the present study. As documented in the literature, AST levels were elevated in 89% of our cases and were consistently higher than ALT levels. Mild transaminase elevations are frequently observed in BACM and are generally interpreted as a biochemical reflection of skeletal muscle injury rather than primary hepatocellular disease, particularly when AST exceeds ALT and both track with CPK dynamics [17,18]. Importantly, markedly elevated ALT levels, a cholestatic pattern, or a lack of parallel improvement with muscle enzymes should prompt evaluation for alternative or concomitant hepatic pathology [19,20]. LDH and myoglobin have also been reported to show transient elevations in patients with BACM, a finding likewise observed in our cohort, further supporting skeletal muscle involvement [14,15].

Regarding hematological findings, leukopenia, with or without neutropenia and/or lymphopenia, and thrombocytopenia were the most common abnormalities. These findings are consistent with previous studies and further support the viral etiology of BACM [1,15].

Azevedo et al. [11] previously identified hospitalization as an independent predictor of higher CPK levels. In our study, higher CPK values were associated with longer hospital stays, suggesting that CPK may serve as a useful biomarker of disease severity. Furthermore, a declining CPK level generally reflects resolution of muscle injury and often parallels symptomatic improvement, supporting an ongoing healing process, as reported in several BACM cohorts [2,5].

In our study, no viral agent was associated with higher CPK values or longer hospital stays, consistent with findings from other studies [1,11]. Nevertheless, a recent post-pandemic study suggested that influenza-associated BACM may be associated with higher peak CPK levels and more pronounced gait impairment compared with non-influenza-related cases [21].

One of the most striking findings of our study was the complete absence of documented BACM cases during the COVID-19 pandemic (2020–2022), followed by a marked post-pandemic increase. A similar trend was reported by Attainese et al. [1] in a five-year cohort of emergency department admissions from 2018 to 2023. This pattern likely reflects a combination of epidemiological and healthcare-related factors. The widespread adoption of infection control measures, including social distancing and mask use, substantially altered the circulation of respiratory viruses, particularly influenza, the main trigger of BACM. In parallel, changes in healthcare-seeking behavior, such as reduced emergency department attendance and delayed medical consultations, as well as temporary constraints on healthcare services, may have limited case detection. The subsequent resurgence of BACM cases in 2024 further underscores the influence of viral epidemiology and healthcare access on disease incidence and reporting. Despite the higher median CPK levels observed in our cohort, only one patient had myoglobinuria, and none progressed to acute kidney injury. Management is mainly supportive, and adequate hydration is recommended to maintain renal perfusion and promote the clearance of myoglobin and other muscle breakdown products, thereby reducing the risk of pigment-induced acute kidney injury [22,23,24]. This approach is particularly relevant when myoglobinuria is suspected or CPK levels are markedly elevated. In our cohort, early fluid therapy and close clinical monitoring were systematically implemented. All patients fully recovered without complications, supporting the favorable prognosis of BACM reported in the literature [1,14].

The recurrence of BACM has been reported in some studies, generally at low rates, although with notable variability [11,14]. Screening for underlying muscle disorders should be considered in patients with recurrent or severe presentations, particularly when CPK elevations exceed ten times the upper limit of normal. In our study, we identified one recurrent case and one patient with exceptionally high CPK levels (20,321 U/L). Both cases were identified in the most recent year of the study period and remain under investigation to exclude underlying metabolic or neuromuscular disorders.

This study has several limitations that should be acknowledged. First, its retrospective, single-center design and relatively small sample size may limit the generalizability of the findings and introduce potential selection and information bias. Second, our study includes only hospitalized patients, which likely overrepresents more symptomatic cases and does not reflect the true incidence of BACM in the general pediatric population, as many mild cases may be managed in outpatient settings.

Our findings reinforce the typical clinical and laboratory profile of BACM, supporting that the diagnosis can be established based on a history of recent viral illness, followed by acute-onset muscle pain and gait disturbances, in the presence of an otherwise normal neurological examination and elevated CPK levels. All suspected cases should be screened for red flags, including recurrence, CPK elevations greater than ten times the upper limit of normal, abnormal renal function, dark urine, heme-positive dipstick in the absence of red blood cells on microscopy, or confirmed myoglobinuria [10]. Hospitalization should be reserved for patients presenting with these red flags or with an inability to maintain adequate oral hydration [11].

## 5. Conclusions

This study provides a comprehensive nine-year retrospective analysis of BACM, reaffirming its characteristic clinical and laboratory features. BACM remains a self-limiting condition with an excellent prognosis, predominantly affecting school-aged boys and strongly associated with influenza B infections. The seasonal variation and post-pandemic increase in cases highlight the role of viral epidemiology on BACM incidence.

The significant correlation between CPK levels and hospital stay duration suggests a potential role of CPK as a prognostic marker for disease severity. While most cases resolve with supportive care, further investigation is warranted in cases with markedly CPK elevations or recurrent episodes.

Clinicians should maintain a high index of suspicion for BACM in children presenting with acute lower limb pain and gait disturbances following viral illnesses. Improved recognition of its clinical course can aid in early diagnosis, reduce unnecessary investigations, and support appropriate clinical management. Future prospective studies may help clarify predictive markers for more severe presentations and long-term epidemiological trends of BACM in the post-pandemic era.

## Figures and Tables

**Table 1:** Epidemiological, clinical and laboratorial characteristics.

**BACM cases, n**	47
**Gender, n (%)**	
Male	31 (66.0)
Female	16 (34.0)
**Age, years**	
Median (IQR)	7 (2)
**Prodromal symptoms, n (%)**	
Fever	46 (97.9)
Cough	34 (72.3)
Rhinorrhea	30 (63.8)
Odynophagia	14 (29.8)
Diarrhea	5 (10.6)
Vomiting	4 (8.5)
**Presenting symptoms/signs, n (%)**	
Bilateral calf pain	45 (95.7)
Gait abnormality	21 (44.7)
Refusal to walk	28 (59.6)
Generalized myalgia	10 (21.3)
**Time of onset of BACM symptoms, days**	
Median (IQR)	3.0 (1.0)
**Length of hospital stay, days**	
Mean (SD)	3.2 (1.1)
**Viral studies, n (%)**	43 (91.5)
Influenza B	28 (59.6)
Influenza A	9 (19.2)
Influenza B and M. pneumoniae	2 (4.3)
Negative	4 (8.5)
**Laboratory findings**	
CPK (U/L), median (IQR)	4986 (5489)
Myoglobin (ng/mL), median (IQR)	680 (1407)
AST (U/L), median (IQR)	178 (167)
ALT (U/L) median (IQR)	51 (38)
LDH (U/L) median (IQR)	445 (167)
Leukopenia, n (%)	19 (40.4)
Thrombocytopenia, n (%)	18 (38.3)

## Data Availability

The data presented in this study are available on reasonable request from the corresponding author due to ethical and privacy restrictions related to confidential pediatric patient information.

## Abbreviations

The following abbreviations are used in this manuscript:

BACM	Benign acute childhood myositis
CPK	Creatine phosphokinase
CRP	C-reactive protein
LDH	Lactate dehydrogenase
